# SEPHS1 promotes SMAD2/3/4 expression and hepatocellular carcinoma cells invasion

**DOI:** 10.1186/s40164-021-00212-7

**Published:** 2021-02-23

**Authors:** Shu Yang, Hongying Zhang, Hua Yang, Jin Zhang, Jiao Wang, Ting Luo, Yangfu Jiang, Hui Hua

**Affiliations:** 1 Laboratory of Oncogene, National Clinical Research Center for Geriatrics, West China Hospital, Sichuan University, Chengdu, 610041 Sichuan China; 2 Department of Pathology, the Second Affiliated Hospital of Zunyi Medical University, Zunyi, Guizhou China; 3 School of Basic Medicine, Chengdu University of Traditional Chinese Medicine, Chengdu, Sichuan China; 4 Cancer Center, West China Hospital, Sichuan University, Chengdu, Sichuan China; 5 Laboratory of Stem Cell Biology, West China Hospital, Sichuan University, Chengdu, 610041 Sichuan China; 6 Department of Abdominal Oncology, the Second Affiliated Hospital of Zunyi Medical University, Zunyi, Guizhou China

**Keywords:** Cancer, SEPHS1, SMAD, TGF-β

## Abstract

**Background:**

Hepatocellular carcinoma (HCC) is one of the common cancers that are very aggressive. The secreted cytokine transforming growth factor-β (TGF-β) promotes cancer metastasis by multiple mechanisms such as epithelial-mesenchymal transition and immune evasion. The canonical TGF-β signaling is largely mediated by smooth muscle actin/mothers against decapentaplegic (SMAD) proteins. The current study aims to explore the regulation of TGF-β/SMAD signaling by selenophosphate synthetase 1 (SEPHS1).

**Methods:**

Immunohistochemistry was used to detect the expression of SEPHS1 in HCC and adjacent liver tissues. Western blotting and quantitative reverse-transcription PCR were used to detect the protein and mRNA levels in HCC cell lines. Cell migration and invasion were determined by transwell assay. Bioinformatic analysis was conducted to determine SEPHS1 expression in HCC and its correlation with the survival of HCC patients.

**Results:**

Here we report that SEPHS1 is a positive regulator of SMAD proteins. SEPHS1 expression is up-regulated in HCC compared with adjacent liver tissues. SEPHS1 knockdown leads to decreased expression of SMAD2/3/4 and mesenchymal markers including snail, slug and N-cadherin in HCC cells. Furthermore, SEPHS1 knockdown results in a decrease in HCC cells migration and invasion, and suppresses the stimulation of HCC cells migration and invasion by TGF-β. Overexpression of SEPHS1 in HCC cells promotes cell invasion, which can be abrogated by SMAD3 knockdown. Lastly, higher expression of SEPHS1 is correlated with poor prognosis in HCC patients, as manifested by decreased overall survival and disease-free survival.

**Conclusions:**

SEPHS1 is a positive regulator of TGF-β/SMAD signaling that is up-regulated in HCC. Increased SEPHS1 expression may indicate poor prognosis for patients with HCC.

## Background

Liver cancer is one of the common cancers and a major cause of cancer-related mortality worldwide. Although the treatment of liver cancer patients has been progressing, the prognosis of patients with liver cancer, especially advanced liver cancer, remains to be very poor. Hepatocellular carcinoma (HCC) is the major type of liver cancer. Hepatitis virus infection and liver fat are major risk factors for liver fibrosis and HCC [1]. The development of HCC is a complex process involving genetic and epigenetic events, and activation of many protein kinases [2, 3]. Mutations of the tumor suppressor gene *TP53* and the oncogene *CTNNB1,* and focal amplifications of *TERT* are part of the genetic drivers that contribute to the development and progression of HCC [4, 5]. In addition, epigenetic silencing of tumor suppressor genes such as *SMPD3* and *NEFH* is detected in HCC [6]. A recent integrative analysis of data from 377 HCC patients identifies 296 protein-coding genes and 88 miRNAs as HCC drivers [7]. These drivers are enriched in multiple pathways such as cell cycle, Wnt signaling, transforming growth factor-β (TGF-β) signaling and JAK-STAT signaling [7].

TGF-β is a pleiotropic growth factor that has diverse roles in epithelial-mesenchymal transition (EMT), development, carcinogenesis, cancer metastasis and immune escape [8–11]. Although TGF-β may inhibit tumorigenesis at the early stage by inducing cell cycle arrest, it stimulates EMT and cancer metastasis at later stage [12]. The canonical TGF-β singaling is mediated by SMADs. Upon TGF-β binding to its receptor complex including type I and type II TGF-β receptors (TGFBR1 and TGFBR2), TGFBR is phoshorylated and activated, and then induces SMAD2/3 phosphorylation. Subsequently, SMAD4 is recruited to the phosphorylated SMAD2/3 complex and translocated into the nucleus, where SMAD3 directly binds to DNA and regulates the transcription of many effector genes [9]. Except for the canonical TGF-β signaling, TGF-β may promote the activation of other signaling pathways, such as phosphoinositide 3-kinase (PI3K) and mitogen-activated protein kinase (MAPK) signalling cascades [12]. Notably, there is cross-talk between the canonical and non-canonical TGF-β signaling pathways. The mechanisms underpinning TGF-β signaling in cancer are quite complex. Given the importance of TGF-β signaling in tumorigenesis, it is crutial to identify the regulators of TGF-β signaling that are aberrantly expressed in human cancer.

Selenophosphate synthetase (SEPHS) is an enzyme that synthesizes selenophosphate, the active selenium donor for selenoproteins and selenium-modified tRNA [13]. There are two mammalian SEPHS paralogues, SEPHS1 and SEPHS2. While SEPHS2 is known to be able to catalyse the synthesis of selenophosphate, it is inconclusive whether SEPHS1 can catalyse the synthesis of selenophosphate [14, 15]. SEPHS1 has an essential role in cell proliferation and survival during embryogenesis [16]. SEPHS1 knockout in *Drosophila* and mouse leads to embryonic lethality [14]. Depletion of SEPHS1 in mouse embryonal carcinoma cells reduces the expression of glutathione system proteins, compromises the redox homeostasis and reverses some of the malignant phenotypes, suggesting that SEPHS1 may be involved in tumorigenesis [14]. A recent bioinformatic analysis suggests that some selenoproteins may be aberrantly expressed in a variety of human cancers [17]. In the current study, we analyze the expression of SEPHS1 in human HCC, and investigate its function in TGF-β signaling. Our data demonstrate that SEPHS1 promotes SMAD2/3/4 expression in HCC cells, and stimulates TGF-β-induced HCC cells migration and invasion. Overexpression of SEPHS1 negatively correlates with the overall survival and disease-free survival of HCC patients.

## Materials and methods

### Cell culture

HepG2 and SK-HEP-1 cells were obtained from Cell Lines Bank, Chinese Academy of Science. The cells were routinely cultured in Dulbecco’s modified Eagle’s medium (DMEM) containing high glucose with 10% fetal borine serum, 100 mg/mL penicillin G, and 50 g/mL streptomycin at 37 °C in a humidified atmosphere containing 5% CO_2._

### Reagents and antibodies

TGF-β was purchased from PeproTech Inc. (Rocky Hill, NJ, USA). Antibodies against SEPHS1 (cat. no. 16635-1-AP, 1:1,000), SMAD2 (cat. no. 12570-1-AP, 1:1,000); snail (cat. no. 13099-1-AP, 1:1,000); SMAD4 (cat. no. 10231-1-AP, 1:1,000) and β-actin (cat. no. 20536-1-AP, 1:1,000) were purchased from Proteintech (Rosemont, IL, USA). Antibodies against SMAD3 (cat. no. CY5013, 1:1,000) and N-cadherin (cat. no. CY5010, 1:1,000) were from Abways Technology Inc. (Shanghai, China). Antibody against slug (cat. no. 9585S, 1:1,000) was purchased from Cell Signaling Technology (Danvers, MA, USA). All siRNAs were custom-synthesized products of GenePharma (Shanghai, China). The target sequences for SEPHS1 knockdown are as follows: 5′- AGGUGUCGUUUGUAAUUCA-3′ for siSEPHS1#1, and 5′-CAGAUUACAUUUACCCGAU-3′ for siSEPHS1#2. The target sequences for SMAD3 knockdown is 5′-GGAGAAAUGGUGCGAGAAG-3′. The negative control (siControl) was purchased from GenePharma (Shanghai, China). The 3FLAG-tagged SEPHS1 expression plasmid (GV141-SEPHS1) was ordered from GeneChem (Shanghai, China).

### Transfection

For transient transfection of plasmids, cells were transfected using Lipofectamine 2000 (Thermo Fisher Scientific, Waltham, MA, USA). For transfection of siRNA, proliferating cells in 6-well plates were incubated with in serum-free DMEM containing Lipofectamine 2000. 4–6 h later, cells were incubated in complete DMEM for 48 h, followed by further experiments.

### Quantitative reverse-transcription PCR analysis

Cellular RNAs were extracted using the Cell Total RNA Isolation Kit (Foregene, Chengdu, China). Subsequently, cDNAs were prepared with oligo dT primers, using the HiScript Q RT SuperMix (Vazyme Biotech, Nanjing, China). Quantitative real-time PCR assay was carried out using the SYBR Master Mix (Vazyme Biotech). The primer sequences are as follows: *SEPHS1*, 5′-CCAGGAGGCGATGATGAACA-3′ (forward) and 5′-AACGACACCTCGTTCCTCTG-3′ (reverse); *SMAD2*, 5′-GTCCATCTTGCCATTCACGC-3′ (forward) and 5′-TTCCTGCCCATTCTGCTCTC-3′ (reverse); *SMAD3*, 5′-TCTGCGTGAATCCCTACCAC-3′ (forward) and 5′-TTTTCGGGGATGGAATGGCT-3′ (reverse); *SMAD4*, 5′-GCGTCAGTGTCATCGACAGA-3′ (forward) and 5′-GTCTTGGGTAATCCGGTCCC-3′ (reverse); *β-actin*, 5′-CAAGGCCAACCGCGAGAA-3′ (forward) and 5′-CCCTCGTAGATGGGCACAGT-3′ (reverse). The levels of *SMAD2/3/4* mRNA normalized to *β-actin* were given by 2-△*Ct,* where △*Ct* is *Ct* (*SMAD2/3/4*)-*Ct* (*β-actin*).

### Western blotting

Total protein was extracted using RIPA buffer supplemented with PMSF, aprotinin, and phosphatase inhibitors cocktail. Protein concentrations were measured with BCA Protein Assay (Thermo Fisher Scientific). 30 μg proteins were separated on SDS-PAGE, transferred onto PVDF membrane and incubated with primary antibodies and appropriate horseradish peroxidase (HRP)-secondary antibodies. Detection was performed with BeyoECL Plus (Beyotime, Jiangsu, China). Images were gathered by Fusion Fx (Vilber Lourmat) imaging system (Marne-la-Vallée, France).

### Cell migration and invasion assay

Cell migration was determined by transwell assay. HepG2 or SK-HEP1 cells (5 × 10^4^) transfected with the indicated siRNA or plasmid were seeded in the upper compartment with 500 μL of serum-free medium containing 2 μg/mL mitomycin C to inhibit cell proliferation, and the lower chamber was filled in 500 μL of DMEM. After 48 h of incubation at 37 °C, the cells on the upper surface of the filter were removed, and the filters were incubated with 100% methanol for 2 min. Migrated cells on the lower side of the filter were stained with 0.5% crystal violet for 20 min, and images were captured using a microscope, followed by analysis of migrated cells on the filter using Image Pro Plus 6.0. software. Cell invasion was also determined by the procedure similar to detecting cell migration. The difference was that cells were seeded in transwell chambers with Matrigel (BD Biosciences) covered. Each chambers were covered with 60 mL Matrigel diluted with DMEM to a certain percentage and incubated at 37℃ for 1 h.

### Immunohistochemical analysis of SEPHS1 in tissue samples

A total of 13 pairs of HCC and adjacent liver tissues were obtained from the Second Affiliated Hospital of Zunyi Medical University (Zunyi, China). All patients underwent surgery between 2019 and 2020. The diagnosis of HCC was confirmed by pathological assessment. The age of patients ranged from 31 to 69 years, with a median age of 50 years. Immunohistochemistry (IHC) was performed on tissue sections from formalin-fixed and paraffin-embedded tissue blocks. Briefly, histologic sections were mounted on slides and deparaffinized followed by rehydration. After the antigen retrieval, the slides were incubated with anti-SEPHS1 antibody overnight at 4 °C, followed by incubating with biotin-labeled secondary antibody for 15 min, and then incubating with streptavidin/HRP for 15 min. The slides were further stained with diaminobenzidine chromagen, and counterstained with hematoxylin. SEPHS1-positive cells were stained brown.

The levels of SEPHS1 expression were scored by both the positive percentage and intensity. The scoring of SEPHS1 positive percentage *(a)* was designated as follows: 0 for a positive percentage less than 5% cells, 1 for a positive percentage between 5 and 25%, 2 for a positive percentage between 25 and 50%, 3 for a positive percentage between 50 and 75%, and 4 for a positive percentage more than 75%. The scoring of SEPHS1 intensity *(b)* was as follows: 0 for negative staining, 1 for light yellow staining, 2 for brown-yellow staining, 3 for chocolate brown staining. The final score was calculated as following formula: SEPHS1 score = *a* × *b*. The SEPHS1 expression was considered low or high if the score was less than 5 or more than 5, respectively.

### Bioinformatic analysis

SEPHS1 transcription in HCC from Rossier liver and Rossier liver 2 datasets was analyzed using the Oncomine online database (http://www.oncomine.org) [18]. *SEPHS1* expression in HCC from the Cancer Genome Atlas (TCGA) database was analyzed using the web server UALCAN (http://ualcan.path.uab.edu) [19]. The Gene Expression Profiling Interactive Analysis platform (GEPIA, http://gepia.cancer-pku.cn/) was utilized to analyze the correlation between SEPHS1 and SMAD2/3/4 mRNA levels, and correlation between SEPHS1 expression and the survival of HCC patients in the TCGA database [20]. The survival analysis was based on SEPHS1 mRNA levels, using log-rank test for the hypothesis evaluation. The cut-off for mRNA levels is customizable to allow the stratification of subjects with low or high levels of SEPHS1 mRNA. The TCGA raw data, such as gene expression and clinical-pathological data, are included in both UALCAN platform and the cBioPortal database (http://www.cbioportal.org).

### Statistical analysis

One-way analysis of variance with post hoc tests was used in statistical analysis of mRNA expression, cell migration and invasion. Comparison between two groups were conducted by students’ *t* test. Correlations were determined using Pearson correlation tests. Log-rank test was applied to evaluate the survival of HCC patients. Differences were considered statistically significant if *p* < 0.05.

## Results

### SEPHS1 is overexpressed in HCC

**Fig. 1:**
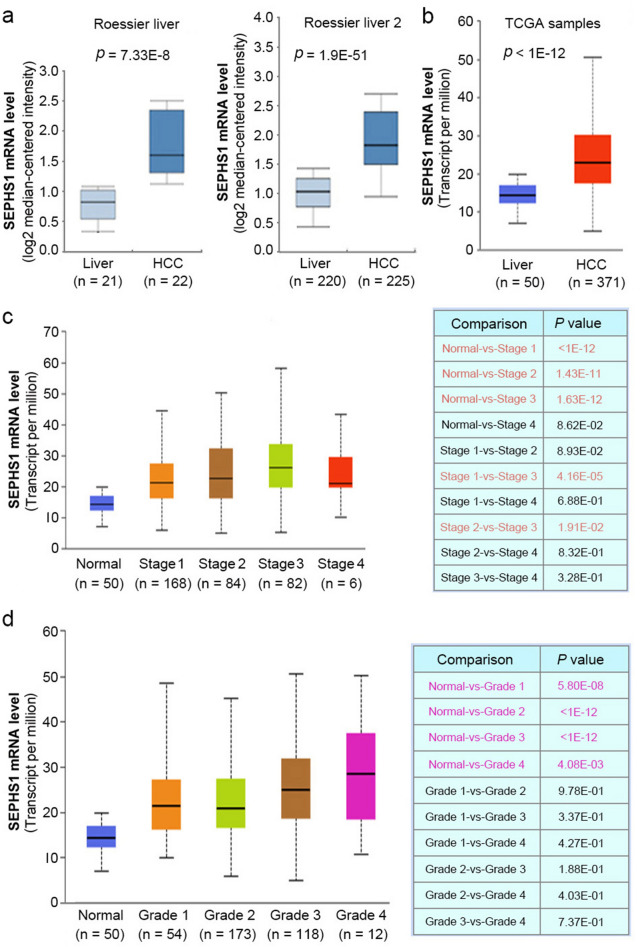
The expression of SEPHS1 mRNA in HCC and adjacent liver tissues. **a** The box plot of SEPHS1 expression in HCC and adjacent liver tissues from Rossier liver and Rossier liver 2 datasets (Oncomine). **b** The box plot of SEPHS1 expression in HCC and adjacent liver tissues from the TCGA project (UCLAN). **c** The SEPHS1 mRNA levels in normal liver tissues and HCC at different stages (Box plot, GEPIA). **d** The SEPHS1 mRNA levels in normal liver tissues and different grades of HCC (Box plot, GEPIA)

To investigate the expression of SEPHS1 in HCC, we analyzed SEPHS1 expression in several HCC datasets. By comparing the mRNA levels of SEPHS1 in 21 normal livers and 22 HCC in the Roessier Liver dataset, we found that the levels of SEPHS1 in HCC were increased in HCC (Fig. 1a). Similar results were detected in the Roessier Liver 2 dataset, which included 220 normal livers and 225 HCC (Fig. 1a). In addition, data from the HCC cohort in the Cancer Genome Atlas (TCGA) database revealed that SEPHS1 is overexpressed in HCC (Fig. 1b). There was significant difference in SEPHS1 expression between normal liver tissues and stage 1/2/3 HCC, and between stage 1/2 and stage 3 HCC (Fig. 1c), while there was no significant difference among different grades of HCC (Fig. 1d). The highest levels of SEPHS1 were detected in stage 3 HCC.

**Fig. 2:**
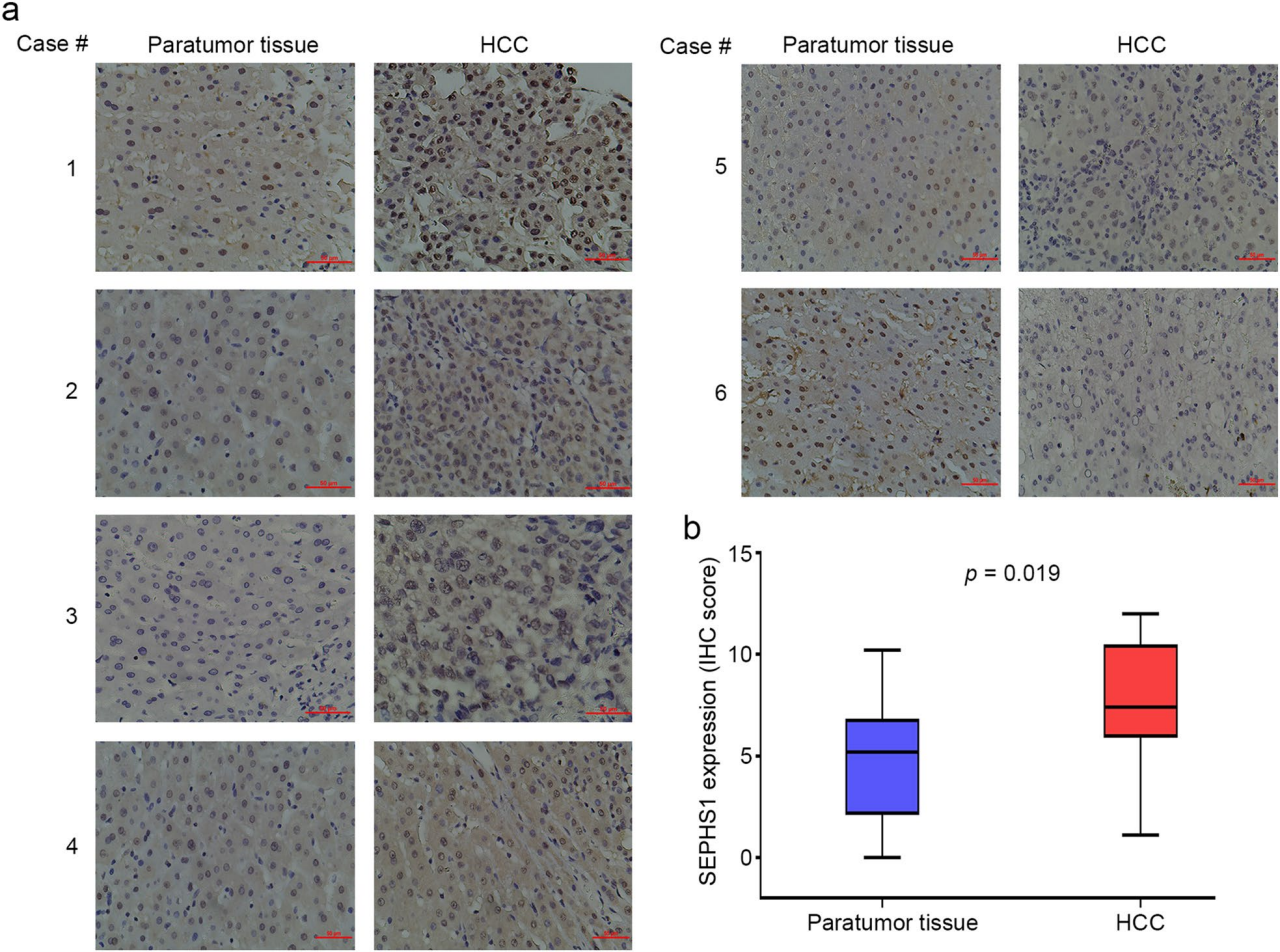
Immunohistochemical analysis of SEPHS1 expression in HCC and paratumor liver tissues. **a** Reprensentative immunohistochemical staining of SEPHS1 in HCC and paratumor tissues. **b** The box plot of SEPHS1 expression in HCC and paratumor liver tissues

In addition, we detected the expression of SEPHS1 protein in 13 pairs of HCC tissues and the adjacent liver tissues. Immunohistochemical analysis demonstrated that SEPHS1 protein was predominantly localized in the nucleus, while it was also distributed in the cytoplasm (Fig. 2). Compared with the adjacent liver tissues, increased expression of SEPHS1 was detected in 53.8% of HCC tissues, similar levels of SEPHS1 expression was detected in 30.8% HCC tissues, and decreased SEPHS1 expression was detected in 15.4% of HCC tissues (Fig. 2). In addition, the percentage of samples with low levels of SEPHS1 expression was much less in HCC than that in adjacent liver tissues (7.7% *vs* 46.2%).

### SEPHS1 promotes SMAD2/3/4 expression in HCC cells

**Fig. 3:**
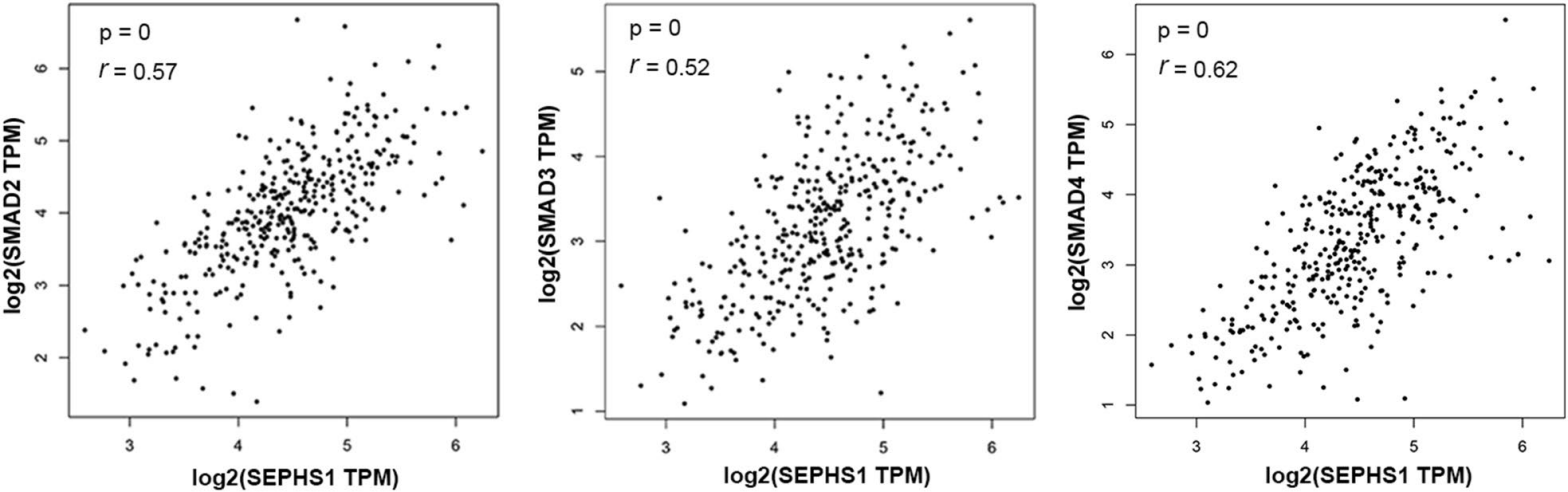
The correlation between SEPHS1 and SMAD2/3/4 mRNA levels in HCC from the TCGA project (GEPIA). Correlations were determined using Pearson correlation tests

**Fig. 4:**
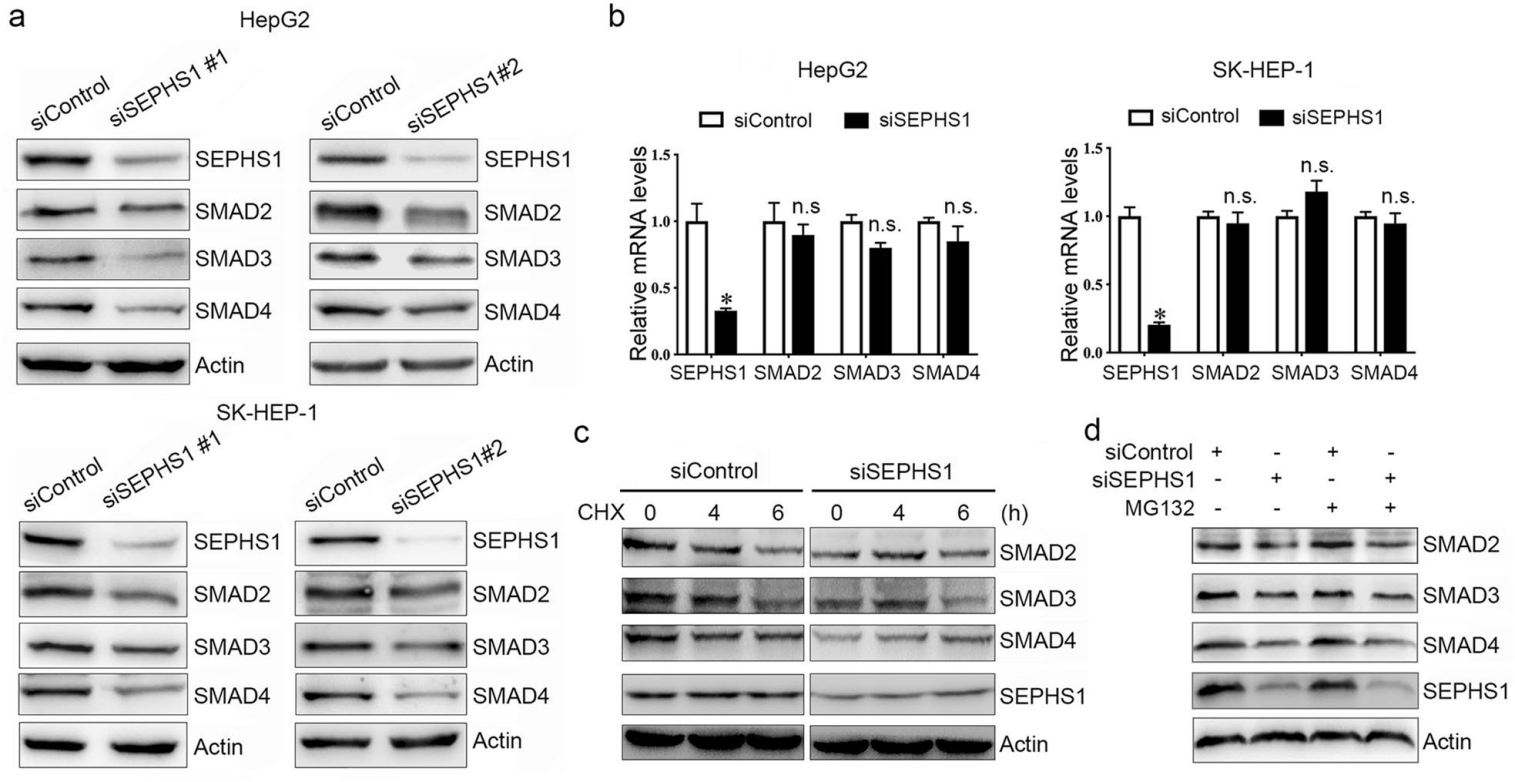
The effects of SEPHS1 knockdown or overexpression on SMAD2/3/4 expression in HCC cells. **a** HepG2 and SK-HEP-1 cells were transfected with siControl, siSEPHS1#1 or siSEPHS1#2, followed by western blot analysis of indicated proteins. **b** HepG2 and SK-HEP-1 cells were transfected with siControl or siSEPHS1, followed by quantitative RT-PCR analysis of indicated mRNA levels. The relative mRNA levels were plotted. *Columns*, mean ± SD (n = 3). *, *p* < 0.01. *n.s.*, not significant. **c** HepG2 cells were transfected with siControl or siSEPHS1 and treated with or without cycloheximide (CHX), followed by western blot analysis of indicated proteins. **d** HepG2 cells were transfected with siControl or siSEPHS1 and treated with or without MG132, followed by western blot analysis of indicated proteins

By analyzing the genes which expression was correlated with SEPHS1 in HCC, we found that the *SEPHS1* mRNA levels were correlated with *SMAD2/3/4* mRNA in the TCGA dataset (Fig. 3). We then detected the effect of SEPHS1 on SMAD2/3/4 expression. Knockdown of SEPHS1 by two different sets of siRNA consistently resulted in decreased expression of SMAD2/3/4 protein in both HepG2 and SK-HEP-1 cells (Fig. 4a). However, quantitative RT-PCR analyses demonstrated that SEPHS1 knockdown did not affect SMAD2/3/4 transcription (Fig. 4b), suggesting that SEPHS1 did not affect SMAD2/3/4 transcription. In addition, treatment with TGF-β had no effect on SEPHS1 expression in HepG2 cells (data not shown). Hence, the positive correlation between *SEPHS1* and *SMAD2/3/4* mRNA levels was not due to mutual regulation. To determine whether SEPHS1 may inhibit the stability of SMAD2/3/4 proteins, HepG2 cells were transfected with SEPHS1 siRNA and treated with the protein translation inhibitor cycloheximide (CHX), followed by western blot analysis of SMAD2/3/4 expression at different time points. While SMAD2/3/4 expression gradually decreased in siControl-transfected HepG2 cells after CHX treatment, the levels of SMAD2/3/4 proteins remained steadily low in SEPHS1 knockdown cells, which did not change significantly even after CHX treatment (Fig. 4c). Moreover, treatment with the proteasome inhibitor MG132 did not rescue SMAD2/3/4 levels in SEPHS1-depleted HepG2 cells (Fig. 4d). Collectively, these data suggest that the upregulation of SMAD2/3/4 expression by SEPHS1 may not be attributed to the regulation of *SMAD2/3/4* transcription and the stabilization of SMAD2/3/4 proteins. It is possible that SEPHS1 may regulate SMAD2/3/4 at the translation level.

### SEPHS1 promotes snail/slug/N-cadherin expression and TGF-β-induced HCC cells migration and invasion

**Fig. 5:**
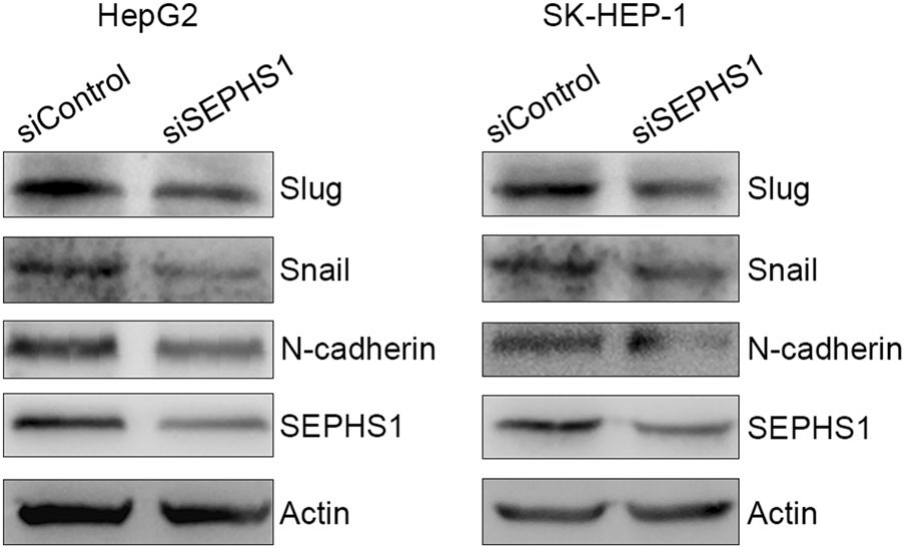
The effects of SEPHS1 knockdown on snail, slug and N-cadherin expression in HCC cells. HepG2 and SK-HEP-1 cells were transfected with siControl or siSEPHS1, followed by western blot analysis of indicated proteins

The canonical TGF-β signaling through SMAD directly or indirectly regulates the expression of EMT-related genes, such as snail, slug and N-cadherin [12, 21]. We then detected the effects of SEPHS1 on the expression of snail, slug and N-cadherin. SEPHS1 knockdown resulted in decreased expression of snail, slug and N-cadherin in both HepG2 and SK-HEP-1 cells (Fig. 5).

**Fig. 6:**
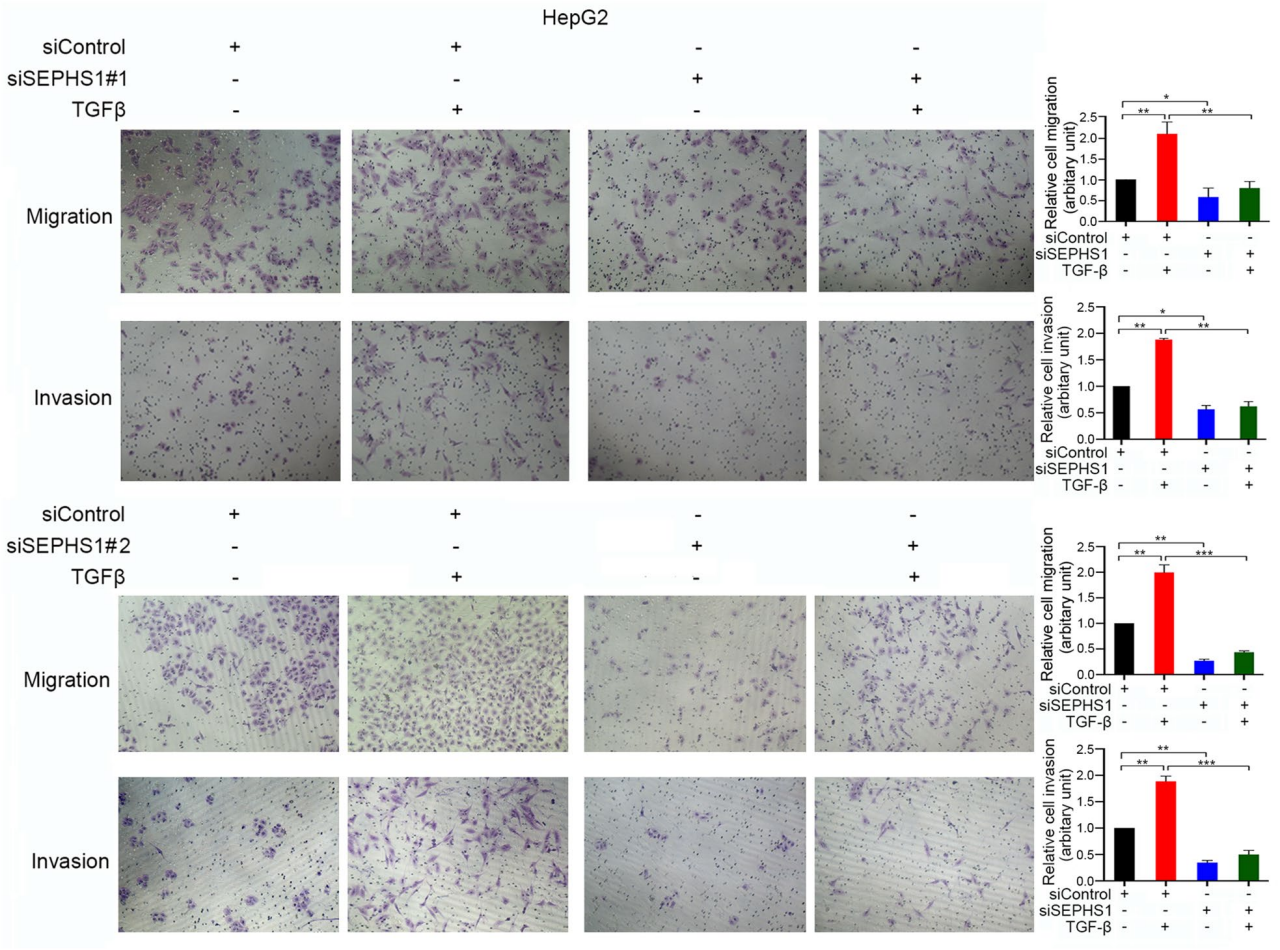
The effects of SEPHS1 knockdown on HepG2 cells migration and invasion. HepG2 cells were transfected with siControl, siSEPHS1#1 or siSEPHS1#2, and treated with or without TGF-β, followed by detection of cell migration and invasion. The relative cell migration or invasion was plotted. Cell migration or invasion in the siControl-transfected and TGF-β-untreated group was set as 1. *Columns*, mean ± SD (n = 3). *, *p* < 0.05. **, *p* < 0.01. ***, *p* < 0.001

**Fig. 7:**
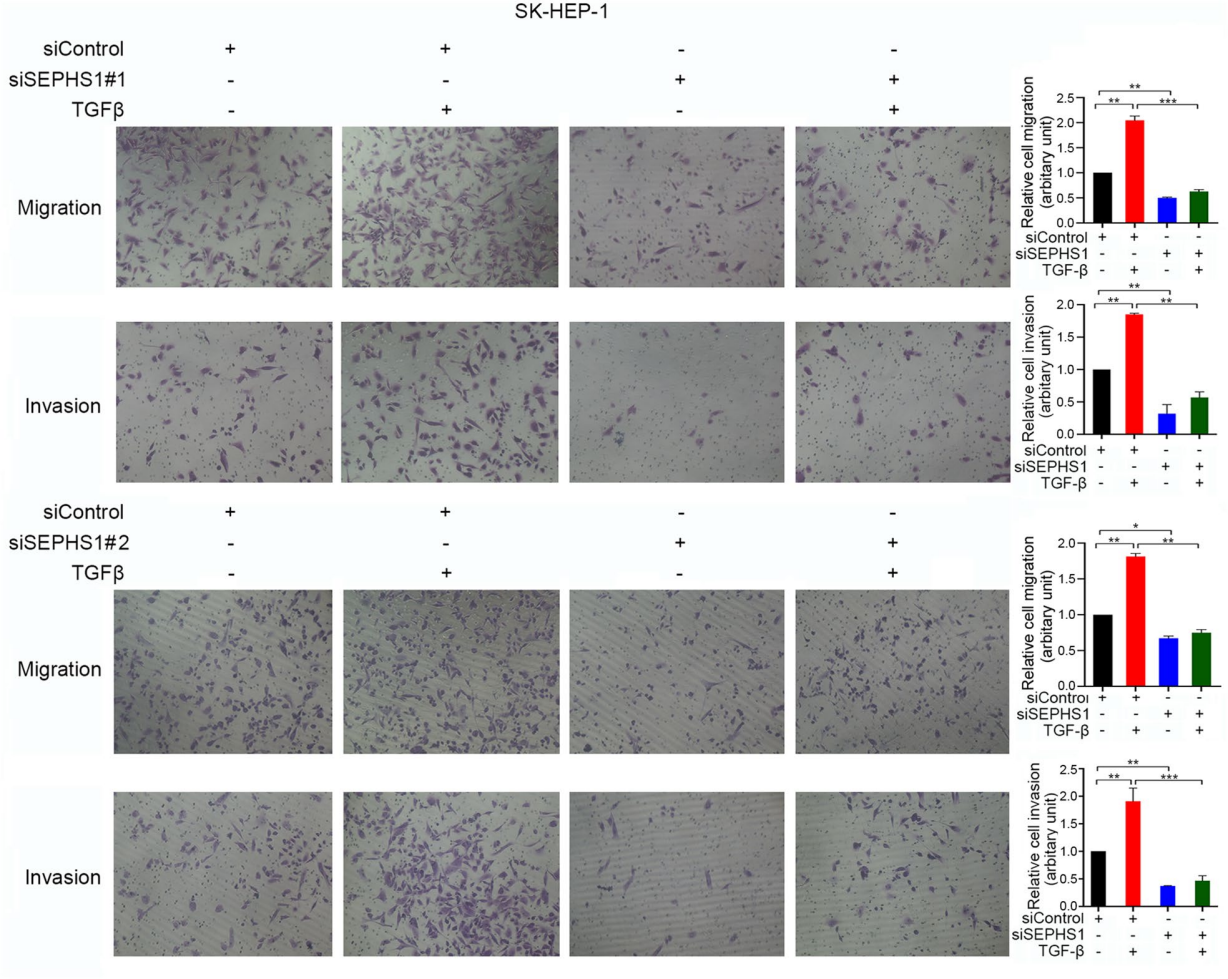
The effects of SEPHS1 knockdown on SK-HEP-1 cells migration and invasion. SK-HEP-1 cells were transfected with siControl, siSEPHS1#1 or siSEPHS1#2, and treated with or without TGF-β, followed by detection of cell migration and invasion. The relative cell migration or invasion was plotted. Cell migration or invasion in the siControl-transfected and TGF-β-untreated group was set as 1. *Columns*, mean ± SD (n = 3). *, *p* < 0.05. **, *p* < 0.01. ***, *p* < 0.001

**Fig. 8:**
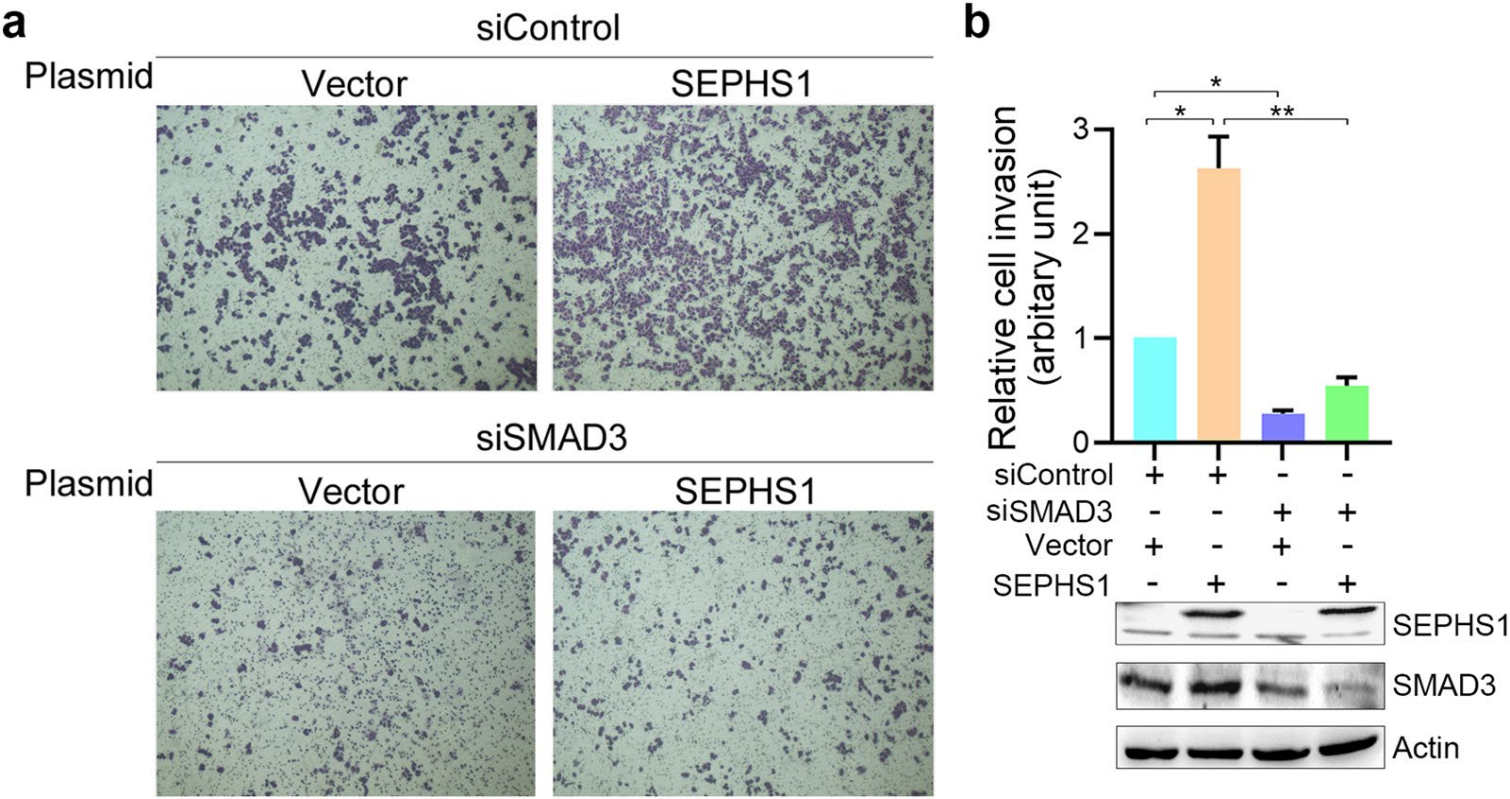
The effects of SEPHS1 overexpression and SMAD3 knockdown on HepG2 cells invasion. **a** HepG2 cells were transfected with empty vector or 3FLAG-tagged SEPHS1 expression plasmid, and siControl or siSMAD3, followed by detection of cell migration and invasion. **b** The relative cell invasion was plotted. Cell invasion in the empty vector- and siControl-transfected group was set as 1. *, *p* < 0.01. **, *p* < 0.001. The efficiency of SEPHS1 overexpression and SMAD3 knockdown was shown

Since TGF-β-induced EMT promotes cancer cell migration and invasion, we then detected the effects of SEPHS1 on TGF-β-induced HCC cells migration and invasion. SEPHS1 knockdown by two different siRNAs consistently inhibited HepG2 cells migration and invasion (Fig. 6). Meanwhile, SEPHS1 knocdown by two different siRNAs consistently suppressed the stimulation of HepG2 cell migration and invasion by TGF-β (Fig. 6). Similar effects were also detected in SK-HEP-1 cells (Fig. 7). In addition, overexpression of SEPHS1 in HepG2 cells promoted cell invasion (Fig. 8). SMAD3 knockdown compromised the promotion of HepG2 cells invasion by SEPHS1 (Fig. 8), indicating that SMAD3 may mediate, at least in part, the promotion of HCC cells invasion by SEPHS1.

### SEPHS1 expression is negatively correlated with the prognosis of HCC patients

**Fig. 9:**
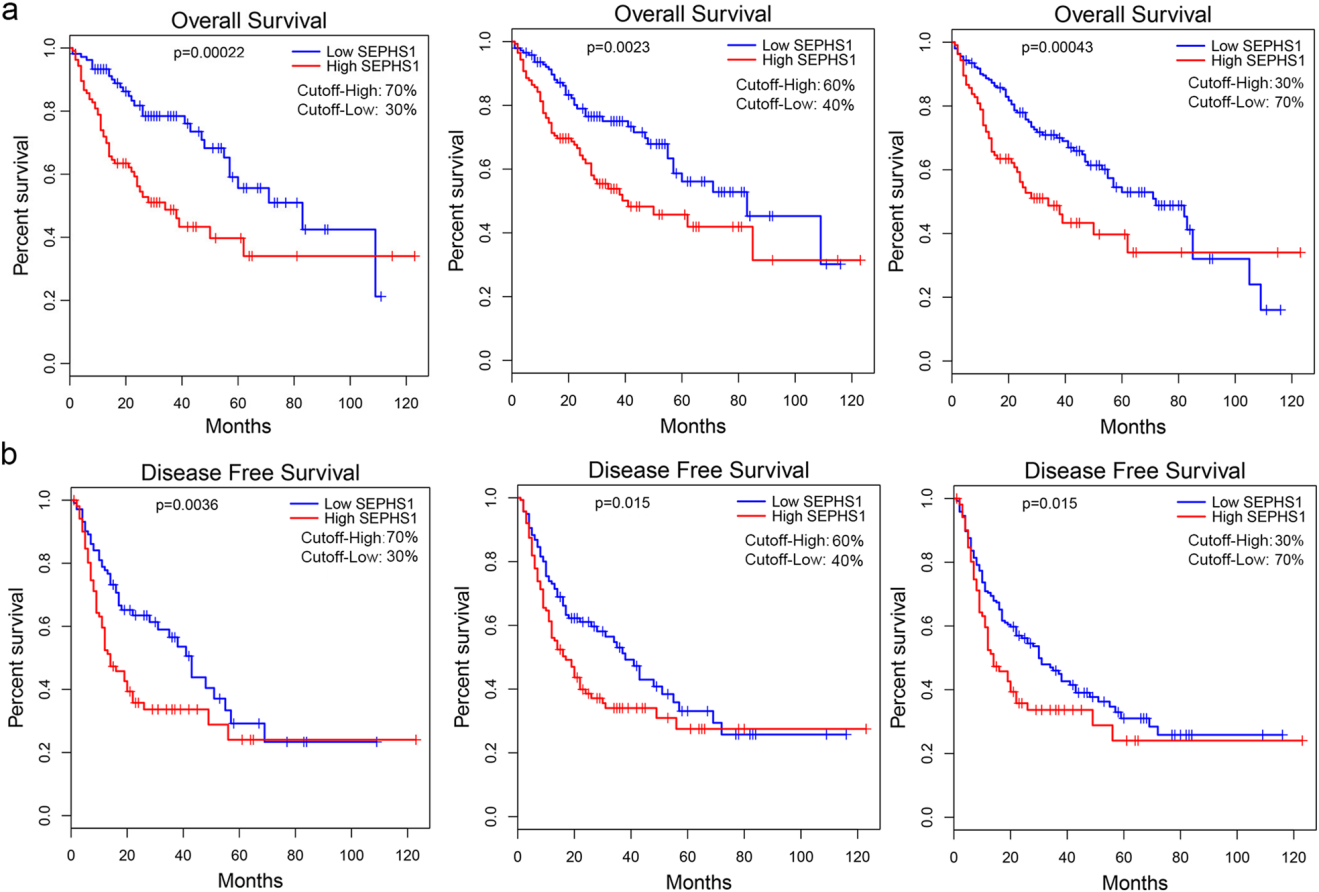
The correlation between SEPHS1 expression and the prognosis of HCC patients from the TCGA project (GEPIA). The Kaplan–Meier survival curves were plotted. Log-rank *p* values were shown. **a** The correlation between SEPHS1 expression and the overall survival of HCC patients. **b** The correlation between SEPHS1 expression and the disease-free survival of HCC patients. A cutoff was set to divide the patients into two groups: low SEPHS1 expression and high SEPHS1 expression. Cutoff-low of 30%, 40% or 70% indicates that 30%, 40% or 70% of patients are assigned into low SEPHS1 expression group, while cutoff-high of 70%, 60% or 30% indicates that 70%, 60% or 30% of patients are assigned into high SEPHS1 expression group

To determine whether SEPHS1 expression is associated with the prognosis of HCC patients, the data from HCC cohort in the TCGA project were analyzed. We found that the levels of SEPHS1 expression was negatively associated with the overall survival in HCC patients at various cutoff, and very significant difference was present when the cutoff-high and cutoff-low were set as 70% and 30%, respectively (Fig. 9a). When the cutoff-high was set as ≥ 30%, there was significant difference in disease-free survival as well, and very significant difference was detected when the cutoff-high and cutoff-low were set as 70% and 30%, respectively (Fig. 9b). These data indicate that SEPHS1 expression negatively influences the prognosis of patients with HCC.

## Discussion

TGF-β signaling in hepatocyte is implicated in liver fibrosis and carcinogenesis. While the C-terminal phosphorylation of SMAD3 by TGFBR1 may be tumor-suppressive, oncogenic linker-phosphorylated SMAD3 signaling is involved in both nonalcoholic steatohepatitis-related HCC and HBV/HCV-related HCC [22–24]. Phosphorylated SMAD3 not only recruits SMAD4 to the nucleus and then regulates target genes transcription, but also stabilize the cytoplasmic β-catenin and help it translocate into the nucleus to promote TCF-mediated genes transcription [25]. TGF-β-SMAD signaling promotes EMT and the metastasis of HCC [26–28]. While the mechanisms of SMAD phosphorylation have been intensively studied, little is known about the regulation of SMAD expression. The cAMP-PKA-CREB signaling may negatively regulate TGF-β signaling by up-regulating the expression of SMAD7 [29, 30]. In addition, the E3 ubiquitin ligase PJA1 is identified as a promoter of SMAD3 degradation [31]. Our current study demonstrates that SEPHS1 is a master regulator of SMAD2/3/4 expression, which is not attributed to the effects on SMAD2/3/4 transcription and the stability of SMAD2/3/4 proteins. It remains to know whether SEPHS1 promotes SMAD2/3/4 translation. However, SEPHS1 mRNA levels seem to correlate with SMAD2/3/4 mRNAs in HCC. It warrants further research to determine whether SEPHS1 and SMAD2/3/4 transcription are regulated by some common nodes, or this correlation is just serendipity. Although SEPHS1 resides in the selenium metabolism pathway, the regulation of SMAD2/3/4 expression by SEPHS1 may be independent of selenium, since selenite and knockdown of the selenophosphate synthase 2 do not affect SMAD2/3/4 expression (data not shown).

As a transcription factor, SMAD3 may regulate the expression of many genes, either positively or negatively. The best-known SMAD3 targets are EMT-related genes such as twist1, snail and slug [8, 12]. EMT is tightly involved in cancer metastasis. The current study demonstrates that SEPHS1 positively regulates snail, slug and N-cadherin expression in HCC cells. Consistent with the promotion of cancer cells migration and invasion by TGF-β/SMAD signaling, SEPHS1 promotes HCC cell migration and invasion. SEPHS1 knockdown abrogates TGF-β-induced HCC cells migration and invasion. On the other hand, the stimulation of HCC cells invasion by SEPHS1 overexpression can be suppressed by SMAD3 knockdown. These data demonstrate that SMAD3 may contribute, at least in part, to the promotion of HCC cells invasion by SEPHS1.

In addition, TGF-β promotes the generation of hepatic tumor-initiating cells during hepatocarcinogenesis [32]. Tumor stemness, invasion and metastasis eventually lead to poor prognosis. Indeed, SEPHS1 expression is associated with poor prognosis in HCC patients. SEPHS1 may be a predictive biomarker for HCC prognosis. Given the promotion of HCC cell migration and invasion by SEPHS1, SEPHS1 may be a therapeutic target of HCC. Targeting TGF-β signaling for cancer therapy has attracted considerable attention because TGF-β has diverse pro-tumor roles [33]. Except for the canonical SMAD signaling, TGF-β not only induces canonical signaling through SMAD, but also triggers noncanonical kinase cascades, leading to the activation of other well-conserved signaling pathways such as PI3K, MAPK and mTOR, which play important roles in tumorigenesis [34, 35]. It warrants further research to determine whether SEPHS1-overexpressed HCC may respond to TGF-β signaling-targeted treatment.

## Conclusions

Our study is the first to demonstrate that SEPHS1 is a positive regulator of SMADs and TGF-β-induced migration and invasion. Aberrant expression of SEPHS1 in HCC may promote the aggresive behavior of HCC cells. The expression of SEPHS1 is a potential prognostic factor for HCC. A better understanding of the functions of SEPHS1 in different types of human cancer may provide more insights into cancer progression.

## Data Availability

The data for the current study are available from the corresponding author on reasonable request. Data from the publicly available datasets used in this study can be accessed at: TCGA (https://www.cancer.gov/tcga), GEPIA (http://gepia.cancer-pku.cn/).

## Abbreviations

EMT: Epithelial-mesenchymal transition
HBV: Hepatitis B virus
HCC: Hepatocellular carcinoma
HCV: Hepatitis C virus
MAPK: Mitogen-activated protein kinase
PI3K: Phosphoinositide 3-kinase
SEPHS1: Selenophosphate synthetase 1
SMAD: Smooth muscle actin/mothers against decapentaplegic
TGF-β: Transforming growth factor-β
TGFBR: TGF-β receptors
